# 

**Published:** 1889-06

**Authors:** 


### CONTENTS:

Article I.—The New Epoch in Medicine.
By Robert Ormiston, M. D. . . . . . 49
" II.—Neurectomy of the Inferior Branch of the Fifth Nerve. By J. H. Glass, M. D. . . . . . 65
" III.—Crown and Bridge Work.
By J. Marion Edmunds. . . . . . 67
" IV.—Sensitive Dentine.
By J. B. Willmott, L. D. S., D. D. S., M. D. S. 78
The National Association of Dental Examiners. . . . . 86
Comments on the New Edition (12th) of Harris' Principles and Practice of Dentistry. . . . . . . 87
The Johnstown Calamity. . . . . . . . . . 90

### EDITORIAL.

University of Maryland Dental Department. . . . . . 91
Charles Wilson Peale as an Inventor of Porcelain Teeth. . 91
Department of the Interior, Census Office. . . . . 94
An Entertaining Periodical. . . . . . . . . 95

### MONTHLY SUMMARY.

Influence of Certain Medicinal Agents on the Bacillus of Tubercle in Man. . . . . . . . . 96

